# Identification and *in vivo* Efficacy Assessment of Approved Orally Bioavailable Human Host Protein-Targeting Drugs With Broad Anti-influenza A Activity

**DOI:** 10.3389/fimmu.2019.01097

**Published:** 2019-06-05

**Authors:** Theresa Enkirch, Svenja Sauber, Danielle E. Anderson, Esther S. Gan, Dimitar Kenanov, Sebastian Maurer-Stroh, Veronika von Messling

**Affiliations:** ^1^Programme in Emerging Infectious Diseases, Duke-NUS Medical School, Singapore, Singapore; ^2^Veterinary Medicine Division, Paul-Ehrlich-Institut, Langen, Germany; ^3^Biomolecular Function Discovery Division, Bioinformatics Institute, Agency for Science, Technology and Research, Singapore, Singapore; ^4^Department of Biological Sciences, National University of Singapore, Singapore, Singapore

**Keywords:** influenza A virus, drug repurposing, antiviral (H1N1 and H3N2) activity, animal models, host protein-targeting drugs

## Abstract

The high genetic variability of influenza A viruses poses a continual challenge to seasonal and pandemic vaccine development, leaving antiviral drugs as the first line of defense against antigenically different strains or new subtypes. As resistance against drugs targeting viral proteins emerges rapidly, we assessed the antiviral activity of already approved drugs that target cellular proteins involved in the viral life cycle and were orally bioavailable. Out of 15 candidate compounds, four were able to inhibit infection by 10- to 100-fold without causing toxicity, *in vitro*. Two of the drugs, dextromethorphan and ketotifen, displayed a 50% effective dose between 5 and 50 μM, not only for the classic H1N1 PR8 strain, but also for a pandemic H1N1 and a seasonal H3N2 strain. Efficacy assessment in mice revealed that dextromethorphan consistently resulted in a significant reduction of viral lung titers and also enhanced the efficacy of oseltamivir. Dextromethorphan treatment of ferrets infected with a pandemic H1N1 strain led to a reduction in clinical disease severity, but no effect on viral titer was observed. In addition to identifying dextromethorphan as a potential influenza treatment option, our study illustrates the feasibility of a bioinformatics-driven rational approach for repurposing approved drugs against infectious diseases.

## Introduction

Influenza A viruses are one of the most important respiratory pathogens. Annual epidemics represent an important disease burden and cause an estimated 250,000–500,000 deaths worldwide, and occasional pandemics are associated with increased morbidity and mortality (1, 2). Influenza A viruses belong to the *Orthomyxoviridae* family and have a segmented negative-sense RNA genome (3). Their envelope contains the ion channel forming M2 protein and the hemagglutinin (HA), and neuraminidase (NA) glycoproteins. Based on the antigenic properties of these viral glycoproteins, influenza A viruses are classified into different subtypes. To date, 18 hemagglutinin (H1–H18) and 11 neuraminidase (N1–N11) subtypes have been identified (3, 4).

With the exception of bat-associated subtypes (4), all influenza A virus subtypes can be found in wild aquatic birds, which are their natural reservoir. From these animals the virus can spread to domestic poultry or directly to humans and other mammalian hosts (5). Pandemics occur if such a new subtype acquires the ability to infect and transmit in the human population. Over the last 20 years, there have been regular introductions of H5N1 strains and occasional cases of H7N1 and H9N2 infections, mostly associated with outbreaks in poultry (6, 7). Since March 2013, human infections with a previously undescribed H7N9 virus were observed, which also circulates in domestic birds without causing severe disease (8). Even though the production of influenza vaccines is well established, and the regulatory process allows for rapid strain update or exchange, it takes 4–6 months until a vaccine against a newly emerging subtype is available in sufficient quantities (2, 9).

Thus, antiviral drugs are an essential component of pandemic response scenarios and play an important role in reducing disease severity during seasonal influenza epidemics. Two classes of approved drugs against influenza A virus infections have been available for years: adamantane-based M2 ion channel blockers, which prevent acidification of the endosome and therefore release of the viral particles into the cytosol (10), and neuraminidase inhibitors, which prevent the release of newly formed viral particles from infected cells (11). In both cases, resistant viruses emerged rapidly after the onset of widespread use, and at this time, all circulating human influenza A viruses are adamantane-resistant (12), and the number of neuraminidase inhibitor-resistant viruses is rapidly increasing (13). Earlier this year, baloxavir marboxil, a cap-dependent endonuclease inhibitor, has been licensed in Japan for the treatment of influenza virus infections (14), and favipiravir, an inhibitor of the RNA-dependent RNA polymerase, is in advanced clinical development stages (15), so additional treatment options are becoming available. Repurposed drugs targeting cellular instead of viral proteins are considered a promising complement, on the one hand because of the potential for efficacy against a broader range of viruses using the respective pathway, and on the other hand due to the reduced probability for acquisition of resistance-conferring mutations without fitness cost (16, 17).

Whole genome siRNA screens have yielded a list of promising candidate proteins and pathways involved in the influenza virus life cycle (18–21). Watanabe et al. previously described a network of 128 consensus human host genes that were identified in at least 2 separate of these experimental screens to be important for the virus life cycle in the host cell (22). In this study, we re-analyzed the network of consensus cellular candidate proteins from these screens and overlapped it with databases of known cellular targets of already licensed drugs, which would thus be immediately available in case of a pandemic. The anti-influenza activity of the top candidates was then first evaluated *in vitro*, followed by an efficacy assessment of the most promising drugs in mice and ferrets.

## Materials and Methods

### Cells and Viruses

Madin-Darby canine kidney (MDCK) cells (ATCC CCL-34) cells were maintained in Dulbecco's modified eagle medium (DMEM) supplemented with 5% fetal calf serum (FCS), and 1% L-glutamine at 37°C. The influenza A virus strains H1N1 A/Puerto Rico/8/34 (PR8, (23), H3N2 A/Victoria/36/2011 (H3N2, kind gift from Ralf Wagner, Paul-Ehrlich-Institut), and H1N1 pdm09A/Mexico/InDRE4487/2009 [H1N1pdm09, (24)] were propagated in MDCK cells cultivated in DMEM supplemented with 0.75 μg/ml tolylsulfonyl-phenylalanyl-chloromethyl-ketone (TPCK)-trypsin (Sigma) at 37°C. After 12–24 h, the virus-containing supernatant was harvested, centrifuged at 4°C for 5 min at 800 *g* to remove cell debris, aliquoted, and stored at −80°C. Virus titers were determined by limited-dilution method and expressed as 50% tissue culture infectious doses per ml (TCID_50_/ml).

### Bioinformatics Analysis

Watanabe et al. previously described a network of 128 consensus human host genes that were identified in at least two separate experimental screens to be important for the virus life cycle in the host cell (22). The 128 gene nodes from this network were originally connected by 431 edges, which constituted the basis for our analysis. We re-analyzed the network and added new edges from co-expression and protein-protein interaction data. For the co-expression, 62 human microarray datasets from NCBI GEO (25) with minimum 25 samples available (to limit to larger studies) were used to search for correlations between pairs of genes. For a given gene, the Pearson correlation coefficient (PCC) is calculated for every other gene over the 62 datasets. PCC >0.5 or smaller than −0.5 was considered for establishing 4,482 co-expression links between gene pairs. Five hundred and seventy-four protein-protein interaction links were derived from our in-house integrated protein interaction database, which is a collection of experimentally determined protein-protein interactions and contains data from 10 publicly available resources (26). 319 virus and gene/protein interactions were retained from the network of 128 consensus human host genes (22). A Perl script was written to format the nodes and edges for GraphViz Version 2.38 [http://www.graphviz.org] and the network drawn with layout from the NEATO spring-based algorithm [http://www.graphviz.org/pdf/neatoguide.pdf]. 14 gene targets of FDA-approved drugs were identified among the 128 nodes by a custom Perl script that identifies direct as well as alternative gene name matches in the DrugBank flat file version 3.0 (27). Fifteen drugs were selected based on oral bioavailability as well as different degrees of connectivity of the respective target genes in our network, price, and low organism toxicity.

### *In vitro* Influenza Virus Inhibition Assays

All compounds were purchased from Sigma and diluted in either water, phosphate buffer saline (PBS), or dimethyl sulfoxide (DMSO, Sigma) to stock concentrations of 0.5 M, and DMEM was used for all further dilutions. For the initial efficacy screen, 12-well plates seeded with MDCK cells were pretreated with the respective compound for 8 h with 50 or 500 μM of each compound. The cells were then washed with PBS, infected with the PR8 at an MOI of 0.01, and cultivated in TPCK-DMEM (0.75 μg/ml) containing the same drug concentration. Sixteen hour post-infection, the supernatant was stored at −80°C, and the amount of virus was quantified by limited dilution method and expressed as 50% tissue culture infectious dose (TCID_50_). To evaluate the inhibitory effect of the lead compounds in more detail, the experiment was repeated including additional drug concentrations and different influenza A strains.

For the calculation of the 50% effective concentration (EC_50_), a modified plaque reduction assay was used (28). Briefly, MDCK cells were seeded in 6-well plates and pretreated for 8 h with the drug of interest at 5-fold decreasing doses. The cells were then infected with the respective virus at a dose that yields 15–45 plaques per well in the absence of treatment. After incubation for 1 h at 37°C, the cells were washed with PBS, and overlayed with avicel (Sigma) containing the respective drug concentration. After 3 days, the overlay was removed and the wells were stained with 1% crystal violet solution (Sigma), and plaques were counted. GraphPad 6 was used for curve fitting and EC_50_ calculation.

### MTT Assay

The MTT assay was performed according to the manufacturer's protocol (Thermo Fisher Vybrant MTT Cell Proliferation Assay Kit). Briefly, MDCK cells grown in a 96-well-plate were treated with the respective drugs at 5-fold descending concentrations starting at 500 mM or left untreated. After 24 h, medium was replaced by PBS containing 1 mM MTT. After incubation for 4 h at 37°C, supernatants were removed, mixed with DMSO and further incubated for 10 min at 37°C before absorption was measured at 562 nm using a PHOmo Microplate reader. To calculate the relative cytotoxicity, the absorbance of untreated cells was set to 1. The 50% cytotoxic concentration (CC_50_) was calculated using GraphPad 6, and the selectivity index (SI) represents the ratio of CC_50_ to EC_50_.

### *In vivo* Efficacy Assessments

All animal experiments were reviewed and approved by the SingHealth IACUC Committee or the German competent authority (Regierungspraesidium Darmstadt) and carried out according to Singaporean or German Animal Welfare Law, respectively. Groups of 4–6 week-old female C57BL/6 mice (Janvier Labs) were anesthetized by intraperitoneal injection of ketamine/xylazine (100/5 mg/kg), followed by intranasal infection with 1 × 10^3^ TCID_50_ of PR8 in 30 μl of DMEM. Based on the tolerated doses published for the respective drug (29), a dose-finding study was performed, resulting in the following doses used in all mouse experiments: 50 mg/kg/day for oseltamivir phosphate, 40 mg/kg/day for naltrexone, 100 mg/kg/day gallium nitrate, 80 mg/kg/day for ketotifen, and 60 mg/kg/day for dextromethorphan. Animals were gavaged once daily starting 1 day before infection. Three days after infection, the mice were sacrificed and the viral titer in the lungs was determined. Toward this, the lung was weighed and homogenized in 500 μl DMEM with 3 × concentrated penicillin/streptomycin using 2 ml homogenizer tubes (Lysing matrix D, MP Biomedicals) and the Precellys 24-Dual Homogenizer (Peqlab). The titer in the supernatants was quantified by limited dilution method on MDCK cells and expressed as TCID_50_/g.

To evaluate the treatment effect on disease severity in a more clinically relevant situation, groups of male, and female adult European ferrets (*mustela putorius furo*, bred in-house) without antibodies against circulating seasonal influenza A virus strains were treated orally every 8 h with either oseltamivir (2.5 mg/kg, Roche) or dextromethorphan (2 mg/kg, Procter, & Gamble) following the manufacturer's dosing and treatment recommendation, starting 24 h before infection, or left untreated. For infection, animals were anesthetized with ketamine and medetomidine (2/0.2 mg/kg), and then inoculated intranasally with 1 × 10^5^ TCID_50_ H1N1pdm09. Treatment was continued until day 4 post-infection. During that time, clinical signs, body temperature, and weight were assessed twice daily, and then daily until day 7. Respiratory signs, sneezing, nose exudates, congestion, and activity were scored using a 0–1–2 scale. Zero indicates minimal deviations of the physiologic state, 1 indicates moderate nasal discharge, congestion, and/or occasional sneezing, and calm demeanor, and 2 indicates severe nasal discharge and/or labored breathing, dyspnea, frequent sneezing, and depression. Nasal washes were collected every day for the first 4 days and on day 7. Toward this, 500 μl of PBS was administered in one nostril and expectorate was collected in 50 ml centrifuge tubes. This procedure was repeated twice to obtain a minimal volume of 400 μl. The virus titer of the nasal washes was determined by limited dilution method.

### Statistical Analyses

Statistically significant differences between animal groups were determined using one-way analysis of variance (ANOVA) with Dunnett's Multiple Comparison Test for mice treatment group comparison and Tukey's Multiple Comparison Test for ferret clinical score comparison using GraphPad 6.

## Results

### Identification of Approved Drugs Targeting Cellular Proteins Involved in the Influenza Virus Life Cycle

**Figure 1:**
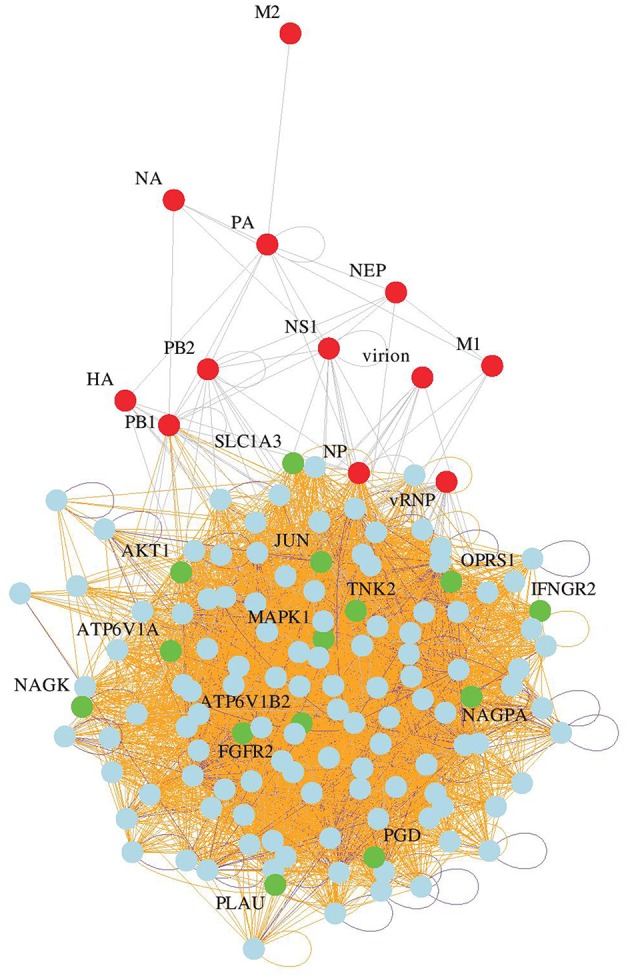
Identification of candidate drugs. Network of influenza host factors with drug targets highlighted. From the network of 128 consensus human host genes (22), 319 virus and gene/protein interactions were retained. Among the 128 nodes, 14 gene targets of FDA-approved drugs were identified by a custom Perl script that identifies direct as well as alternative gene name matches in the DrugBank flat file version 3.0 (27).

Whole genome siRNA screens using different influenza A virus strains and cell lines have yielded a list of cellular proteins that are required in the influenza virus life cycle (22). While it is in most cases unknown if these identified proteins are directly interacting with viral proteins, or if they act indirectly, drugs that modulate their expression levels or activity may also inhibit influenza virus. Therefore, we computationally searched for FDA-approved drugs annotated in DrugBank (27) to target any of the genes in our updated host factor network generated by a meta-analysis of available influenza virus siRNA screen data sets (Figure 1). This bioinformatics analysis yielded 23 candidates, of which 15 drugs linked to 14 different host genes were chosen for further characterization based on oral bioavailability, since this would be a prerequisite for widespread use during a severe epidemic or pandemic. In addition, price, low organism toxicity, commercial availability, and level of connectivity of the respective target genes in our network were also taken into consideration (Table 1).

**Table 1:** Inhibition of influenza A virus replication by licensed drugs with Drug ID and cellular targets identified by bioinformatics analysis.

**Drug Candidate**	**Drugbank ID**	**Annotated Target**	**Inhibitory effect**
Arsenic trioxide	DB01169	AKT1, JUN, MAPK1	Excluded; high organism toxicity
Tiludronate	DB01133	ATP6V1A	Excluded; no oral administration
Palifermin	DB00039	FGFR2	Excluded; no oral administration
Interferon gamma-1b	DB00033	IFNGR2	Excluded; no oral administration; costly
Pentazocine	DB00652	OPRS1	Excluded; controlled substance
Remoxipride	DB00409	OPRS1	Excluded; no longer licensed
Gadopentetate dimeglumine	DB00789	PGD	Excluded; no oral administration
Urokinase	DB00013	PLAU	Excluded; no oral administration
Isoproterenol	DB01064	MAPK1	toxic
Alendronate	DB00630	ATP6V1A	toxic
Vinblastine	DB00570	JUN	toxic
Irbesartan	DB01029	JUN	toxic
Adenosine triphosphate	DB00171	AKT1,TNK2	No inhibition
Etidronic Acid	DB01077	ATP6V1A	No inhibition
N-Acetyl-D-Glucosamine	DB00141	NAGK, NAGPA	No inhibition
L-Glutamic Acid	DB00142	SLC1A3	No inhibition
Thalidomide	DB01041	FGFR2	No inhibition
Amiloride	DB00594	PLAU	No inhibition
Dacarbazine	DB00851	PGD	no inhibition
Gallium nitrate	DB05260	ATP6V1B2	>10-fold inhibition
Naltrexone	DB00704	OPRS1	>10-fold inhibition
Dextromethorphan	DB00514	OPRS1	>10-fold inhibition
Ketotifen	DB00920	PGD	>10-fold inhibition

### Four of the Identified Compounds Inhibit Influenza A Virus Replication

To identify candidates with influenza virus inhibitory activity, cells were pre-treated with the respective drug at 50 and 500 μM, and virus titers were quantified 16 h after infection with PR8, which corresponded to the peak titer in the untreated samples. Four of the compounds, isoproterenol, alendronate, vinblastine, and irbesartan, displayed pronounced toxicity at the 500 μM concentration, and no antiviral activity at a 10-fold lower concentration (Table 1). Adenosine triphosphate, etidronic acid, N-acetyl-D-glucosamine, L-glutamic acid, thalidomide, amiloride, and dacarbazine had no antiviral activity (Table 1). Thus, these 11 compounds were excluded from further evaluation. The four remaining compounds, gallium nitrate, naltrexone, dextromethorphan, and ketotifen, were able to inhibit infection at doses above 50 μM without causing toxicity (Table 1). Gallium nitrate is used to treat cancer-associated hypercalcemia, since it prevents osteoclastic activity, by inhibiting ATPase-dependent proton pumps (30, 31). These pumps, including the vATPase (ATP6V1B2, Table 1) are also involved in acidification of the endosome, which may explain its inhibitory effect on influenza A viruses (32). Naltrexone, a competitive opioid receptor agonist which primarily targets μ- and to a lesser extent the κ-opioid receptors, and dextromethorphan, a σ_1_-receptor, and μ-opioid receptor agonist, can both act on the σ_1_-receptor (OPRS1) (33). OPRS1 is involved in diverse intracellular processes (34), including the modulation of innate and adaptive immune responses (35), which may be the basis for its antiviral activity against influenza. Ketotifen, a histamine H1 receptor blocker (36), was found to block 6-phosphogluconate dehydrogenase (PGD) *in vitro* (37), which is upregulated in influenza A virus-infected cells (38).

**Figure 2:**
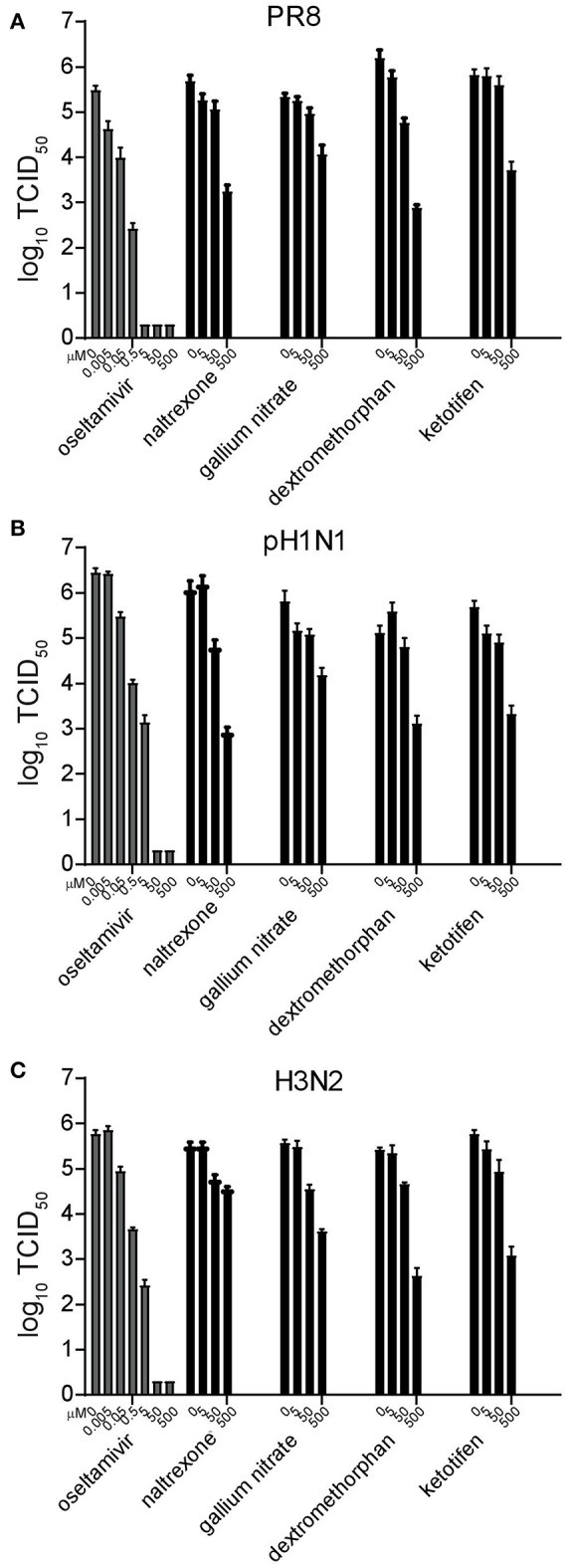
Validation and inhibitory concentration determination of candidate drugs. Replication inhibition of the different drugs. MDCK cells were pretreated for 8 h and infected with the influenza strains A/Puerto Rico/8/34 (PR8) **(A)**, A/Mexico/InDRE4487/2009 (H1N1pdm09) **(B)**, or A/Victoria/36/2011 (H3N2) **(C)** at a MOI of 0.01 in the presence of 10-fold serial drug dilutions. After 16 h, the supernatant was harvested and the amount of virus quantified by limited dilution method. Bars represent the average of three independent duplicate experiments, and error bars indicate the standard deviation.

To determine whether the antiviral activity of these four potential candidates extended to circulating seasonal H3N2 and pandemic H1N1 influenza strains, A/Victoria/36/2011 (H3N2), and A/Mexico/InDRE4487/2009 (H1N1pdm09) were included in the study. The neuraminidase inhibitor oseltamivir carboxylate was used as a positive control. Using the same experimental setup with 10-fold decreasing drug concentrations starting at 500 μM, oseltamivir carboxylate completely inhibited replication of all three strains at concentrations higher than 5 μM (Figure 2). In contrast, all four drugs targeting cellular proteins were only able to reduce viral titers starting at concentrations around 5 μM (Figure 2). This effect ranged from around 10-fold for gallium nitrate to 100-fold for naltrexone and ketotifen, and around 1,000-fold for dextromethorphan. While the extent of inhibition varied for each of the viruses, this tendency was consistently observed, thereby validating our initial findings.

To calculate the EC_50_ for each drug against the different viruses, a more sensitive modified plaque assay was used, which assesses the percent plaque reduction at the respective drug concentration (28). Using 5-fold decreasing drug concentrations, again starting at 500 μM, the EC_50_ of oseltamivir carboxylate ranged between 5 and 200 nM, with PR8 being most effectively inhibited (EC_50_ of 5 nM), while the EC_50_ for H1N1pdm09 was around 200 nM (Table 2). Naltrexone inhibited both H1N1 viruses with an EC_50_ between 5 and 50 μM but was less efficient against the H3N2 virus, whereas gallium nitrate, ketotifen, and dextromethorphan displayed EC_50_ between 5 and 50 μM against all three viruses (Table 2). To determine the selectivity index (SI), the cytotoxic concentration for each of the drugs was determined in MDCK cells using an MTT assay. While the SI for oseltamivir carboxylate was between 2,000 and 100,000, the SI for the drugs targeting cellular proteins ranged between 10 and 1000 with the SI of dextromethorphan being between 50 and 100 across different viruses (Table 2).

**Table 2:** Fifty percent cytotoxic concentration (CC_50_) in MDCK cells, 50% effective concentration (EC_50_) measured by modified plaque reduction assay, and the resulting selectivity index (SI) of the different compounds against H1N1 and H3N2 influenza A viruses.

		**PR8**		**pH1N1**		**H3N2**	
	**CC_**50**_ (μM)**	**EC_**50**_ (μM)**	**SI**	**EC_**50**_ (μM)**	**SI**	**EC_**50**_ (μM)**	**SI**
Oseltamivir	470 ± 12.3	0.005 ± 0.0004	94,000	0.2 ± 0.05	2350	0.05 ± 0.003	9,400
Naltrexone	5233 ± 227	4.7 ± 0.2	1,113	48.0 ± 3.2	109	>500	n.d.
Gallium nitrate	716 ± 116	54.8 ± 2.2	13.1	55.3 ± 2.9	12.9	5.7 ± 0.9	126
Ketotifen	291 ± 14.8	5.9 ± 0.2	49.3	33.7 ± 1.3	8.6	48.5 ± 2.0	6.0
Dextromethorphan	3546 ± 11.1	48.7 ± 2.3	72.8	50.8 ± 3.3	69.8	51.7 ± 4.2	68.6

### Prophylactic Treatment With Ketotifen or Dextromethorphan Reduces Lung Titers in Mice

**Figure 3:**
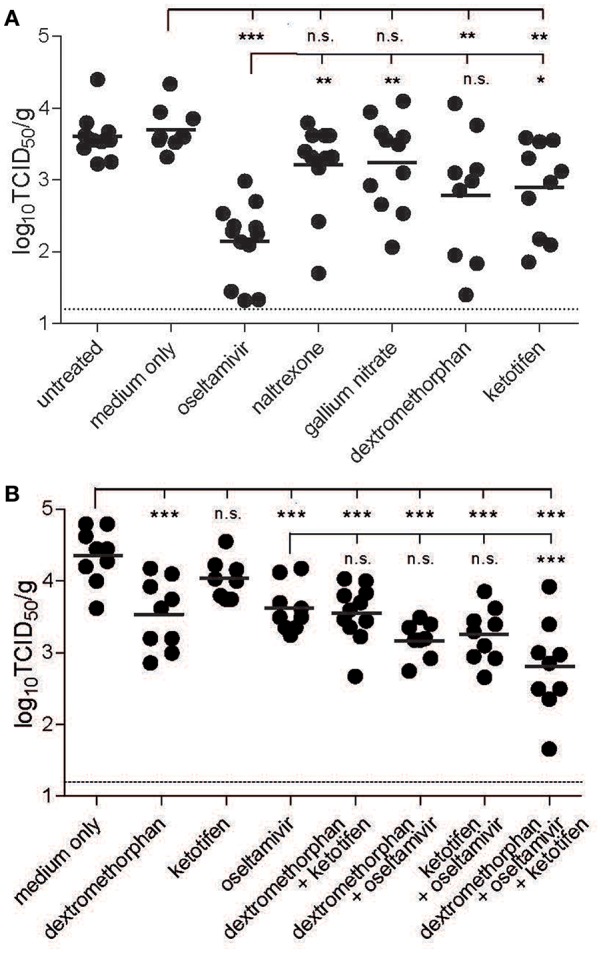
Inhibitory effect of drug candidates in mice. C57BL/6 mice were treated orally once daily starting 1 day before infection and continuing until sacrifice with the respective drug at the maximum tolerated dose individually **(A)** or in combination **(B)**. The animals were infected intranasally with 10^3^ TCID_50_ of PR8. Three days post-infection, the animals were sacrificed and the lung titers were quantified by limited dilution method and expressed as TCID_50_/g tissue. Bars indicate group means, and the dotted line reflects the detection limit of the assay. One-way analysis of variance (ANOVA) with Dunnett's Multiple Comparison Test was used for statistical analyses (no significance (n.s.), ^*^p < 0.05, ^**^p < 0.01, ^***^p < 0.001).

To gain first insights in the ability of the four candidate drugs to reduce virus replication *in vivo*, C57BL/6 mice were treated orally once a day starting 1 day before infection with the respective drug. For all drugs, the initial doses were chosen based on published tolerated doses (29, 39–41), and optimal concentrations were determined in an initial dose-finding study (data not shown). Comparison of lung virus titers at peak infection and survival curves revealed that statistically significant differences could be attained with smaller groups if lung virus titers were measured, since drug treatment delayed but did not completely prevent mortality (Supplemental Figure 1). Toward this, animals were infected intranasally with 10^3^ TCID_50_ PR8, sacrificed 3 days post-infection, and the viral load in the lung was quantified. Oseltamivir phosphate was most effective, reducing lung titers 28-fold, followed by dextromethorphan, which resulted in a more variable but significant reduction. Ketotifen was less effective, but still lowered lung viral titers around 8- to 10-fold, while naltrexone and gallium nitrate treatments had no significant effect (Figure 3A).

As a σ_1_-receptor agonist, dextromethorphan targets a different cellular pathway than ketotifen, which is a histamine H1 receptor blocker (36, 42). To evaluate potential additive effects of the two drugs and oseltamivir, mice were again treated orally once daily with the respective drug combinations, starting again 1 day before infection. In this series of experiments, the inhibitory effect of ketotifen alone was even less pronounced, and there was no additive effect when ketotifen and dextromethorphan were combined. Combination of each of the drugs with oseltamivir resulted in a slight, but non-significant increase in inhibition, but when all three drugs were combined, the inhibitory effect was significantly higher than that of oseltamivir alone (Figure 3B), suggesting a modest benefit of combination treatment in this context.

### Dextromethorphan Has no Effect on Viral Load but Reduces Clinical Disease in Ferrets

To assess the therapeutic potential of dextromethorphan in an animal model that reproduces the course of disease seen in humans (43, 44), ferrets were treated every 8 h with either commercially available oral dextromethorphan formulation at a dose of 2 mg/kg, or oseltamivir solution at a dose of 2.5 mg/kg. This schedule was in accordance with the recommendation for dextromethorphan treatment of patients, and the doses were within the weight adjusted human dose range. Drugs were given for the first time 24 h prior to infection and treatment was continued for 5 days. Nasal washes were collected daily, and the animals were evaluated twice daily for weight loss, temperature, and clinical signs.

**Figure 4:**
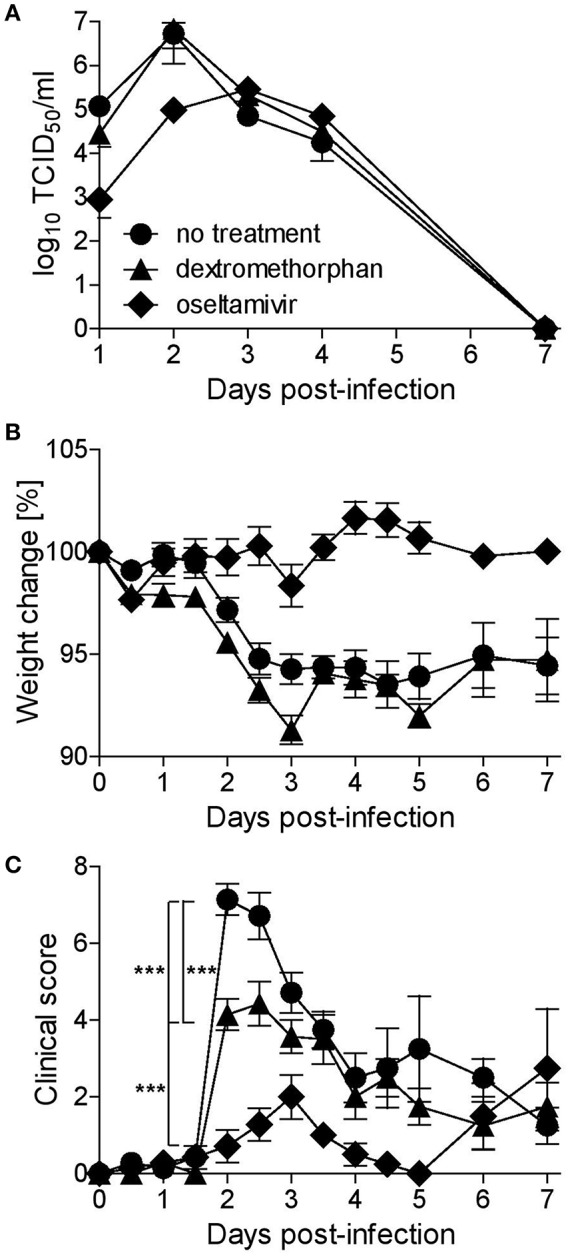
Dextromethorphan efficacy assessment in ferrets. Ferrets (*n* = 9) were treated orally every 8 h for 5 days or until sacrifice on day 3 (*n* = 4), starting 1 day before infection with 2 mg/kg dextromethorphan or 2.5 mg/kg oseltamivir, corresponding to recommended human doses. The animals were infected intranasally with 10^5^ TCID_50_ of A/Mexico/InDRE4487/2009. **(A)** Nasal washes were collected daily, and titers were quantified by limited dilution method. For the first 4 days, animals were monitored twice daily and daily thereafter for weight **(B)** and clinical signs **(C)**. The clinical score represents a composite score of body temperature, activity, nasal exudate, and respiratory signs scored on a 0-1-2 scale with 0 representing body temperatures ≤39.3°C and the physiologic state, 1 temperatures 39.4–39.9°C, calm behavior, low amounts of clear exudate, and occasional sneezing or congestion, and 2 temperatures ≥40.0°C, depressed behavior, larger amounts of yellow or brown exudate, and frequent sneezing, coughing and/or dyspnea. Symbols represent group averages, and error bars indicate the standard error mean. One-way analysis of variance (ANOVA) with Tukey's Multiple Comparison Test was used for statistical analyses (^***^p < 0.001).

While oseltamivir phosphate treatment resulted in a 10-fold reduction in nasal wash titers during the first 2 days, dextromethorphan had no effect (Figure 4A), and none of the treatment approaches affected lung viral titers in animals sacrificed on day 3 (data not shown). In all three groups, the titers peaked on day 2 post-infection and then gradually decreased (Figure 4A). Dextromethorphan-treated animals also experienced a similar gradual weight loss of <10% seen in non-treated control animals, whereas there was no reduction in body weight in the oseltamivir group (Figure 4B). In the control group, clinical signs and fever became first apparent 36 h post-infection, peaked after 48 h, and then gradually decreased (Figure 4C). Dextromethorphan treatment resulted in a significantly lower peak score (Figure 4C). Even though the effect was less pronounced compared to oseltamivir phosphate treatment, which only resulted in a minimal clinical score peaking on day 3 (Figure 4C), it illustrates the potential of repurposing drugs targeting cellular factors involved in the viral life cycle.

## Discussion

Antiviral approaches targeting cellular proteins or pathways involved in the viral life cycle rather than viral proteins directly have great appeal as they are thought to be less prone to resistance development and may be effective against different strains and even different virus families (16, 45). For influenza virus, several whole genome siRNA screens have been published in recent years (18–21), allowing the cross-matching of host proteins required for influenza replication with known cellular drug targets. Using this approach, we identified a short list of 15 FDA-approved candidate drugs, of which naltrexone, gallium nitrate, ketotifen, and dextromethorphan were able to inhibit viral replication *in vitro* in a dose-dependent manner. Successive *in vivo* evaluation in mice revealed that dextromethorphan was the most promising candidate. Dextromethorphan treatment of ferrets infected with a H1N1pdm09 strain had no effect on viral load or body weight, but resulted in a significant reduction of clinical signs, supporting its further consideration as an influenza treatment option.

High-throughput siRNA screens have opened a new avenue for drug repurposing (46). This is of special relevance for infectious disease targets with lesser market potential, as the development of new drugs is time consuming and costly (47, 48). The severe side effects associated with the chemotherapeutics and immune modulators often found among the top hits may not be acceptable in the context of the target disease, so they are usually not evaluated any further. *In vitro* efficacy assessments of the remaining hits are amenable to medium or high throughput screening, allowing a rapid identification of the most promising candidates (49). Here we show that an *in vivo* efficacy assessment of such candidates is warranted even if the EC_50_ is in the μM range and the SI is consequently low, especially if the drug has a broad therapeutic range, as they may be attractive options for treatment in combination with antivirals or other drugs targeting complementary cellular pathways involved in the viral life cycle.

Two of the identified drugs, ketotifen, and dextromethorphan, not only led to a reduction of viral titers *in vitro*, but also in mice. While ketotifen blocks histamine H1 receptors and the release of inflammatory mediators, and is used to treat asthma, rhinitis, skin allergies, and anaphylaxis (36, 50), dextromethorphan is primarily a σ_1_-receptor agonist and used as an alternative to opioid-containing cough suppressants (51, 52). The antiviral properties of ketotifen may thus be associated with its anti-inflammatory effect (39), which could be substantiated by comparatively quantifying the release of inflammatory mediators in ketotifen- and untreated infected cells or animals. In contrast, the mechanism of action is less obvious for dextromethorphan. Its target, the σ_1_-receptor, is a chaperone located at the mitochondria-associated endoplasmic reticulum (ER) membrane (53). The protein plays an important role in cell homeostasis, since its knock down leads to ER stress, induction of the unfolded-protein response (UPR), dysfunctional autophagosomes, and oxidative stress (54–56), and it also modulates Ca^2+^ release through the interaction with inositol 1,4,5-phosphate receptors (IP3R) (57). This modulation may underlie the anti-influenza effect of dextromethorphan, since viral transcription and replication require Ca^2+^ action through calmodulin (20, 58), and Ca^2+^ is also involved in viral entry through the regulation of clathrin-mediated and clathrin-independent endocytosis (59). If the primary mode of action of dextromethorphan-mediated σ_1_-receptor signaling inhibition lies in such a direct inhibition of viral entry or replication, or rather in the modulation of innate immune responses (34) is the subject of ongoing studies.

Ferrets closely mimic the course of influenza disease seen in humans (44, 60). Seasonal and pandemic 2009 influenza viruses thus cause a mild to moderate disease of 3–5 days duration (23, 61), making it challenging to discern modest differences in disease severity and pathological and virological parameters. While oseltamivir reduced viral load and clinical disease, and prevented weight loss, dextromethorphan treatment only reduced disease severity, and seemed to slightly increase the weight loss compared to untreated animals. However, this seemingly small effect may make the difference between seeking medical attention or just staying at home, and may also reduce the days of lost productivity during the annual epidemics. Future studies are warranted to evaluate if combination treatment increases the efficacy of oseltamivir or other virus-targeting drugs, especially when treatment is started after the onset of clinical disease. There may also be room for short-term treatment at higher doses, given that the dosing regimen used here did not exceed recommended human weight-adjusted doses. Considering that dextromethorphan-containing drugs are frequently used as over the counter remedies for influenza and influenza-like respiratory infections, a more detailed evaluation of its efficacy, especially in combination with other influenza-specific drugs, would be warranted.

## Ethics Statement

This study was carried out in accordance with the recommendations of the SingHealth IACUC committee or the county experimental animal oversight agency (Regierungspraesidium Darmstadt), respectively.

## Author Contributions

TE performed most of the experiments. TE and VvM conceived the study, analyzed, and interpreted data, and wrote the manuscript. EG and DA designed and conducted the initial screening experiments. SS assisted with the *in vitro* and animal studies validating the top hits. DK and SM-S performed the bioinformatics analyses and wrote part of the manuscript.

### Conflict of Interest Statement

The authors declare that the research was conducted in the absence of any commercial or financial relationships that could be construed as a potential conflict of interest.
