# Supplementary figures and images for: Identification and in vivo Efficacy Assessment of Approved Orally Bioavailable Human Host Protein-Targeting Drugs With Broad Anti-influenza A Activity
Source: Front Immunol. 2019 Jun 5;10:1097. doi: 10.3389/fimmu.2019.01097 (PMC6563844; doi:10.3389/fimmu.2019.01097)

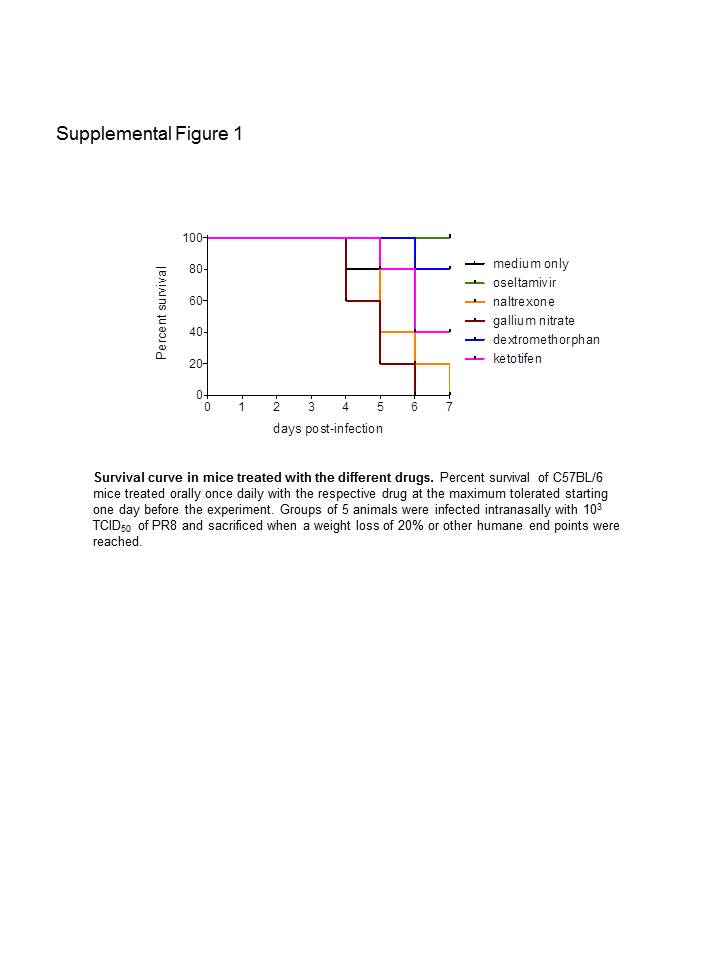

Supplement: Supplementary file 1 [file Image_1.JPEG]
